# Prompt-dependent performance of multimodal AI model in oral diagnosis: a comprehensive analysis of accuracy, narrative quality, calibration, and latency versus human experts

**DOI:** 10.1038/s41598-025-22979-z

**Published:** 2025-10-30

**Authors:** Fatma E.A. Hassanein, Yousra Ahmed, Shaymaa Maher, Ahmed El Barbary, Asmaa Abou-Bakr

**Affiliations:** 1 Oral Medicine, Periodontology, and Oral Diagnosis, Faculty of Dentistry, King Salman International University, El Tor, South Sinai Egypt; 2 Prosthodontics Dentistry, Faculty of Dentistry, King Salman International University, El Tur, South Sinai Egypt; 3 Endodontics Department, Faculty of Dentistry, Galala University, Suez, Egypt; 4 Oral Medicine and Periodontology, Faculty of Dentistry, Cairo University, Giza, Egypt; 5 Oral Medicine and Periodontology, Faculty of Dentistry, Galala University, Suez, Egypt

**Keywords:** Oral diagnosis, Artificial intelligence, Prompt engineering, Multimodal LLMs, Gemini pro, Oral lesions, Histopathology, Diagnostic accuracy, Clinical decision support, Computational biology and bioinformatics, Health care, Mathematics and computing, Medical research

## Abstract

Prompt design is a critical yet underexplored factor influencing the diagnostic performance of large language models (LLMs). Gemini Pro 2.5 shows promise in multimodal reasoning, but no prior study has systematically compared prompt structures in oral datasets against expert benchmarks. This study aimed to evaluate the diagnostic performance of a multimodal LLM (Gemini Pro 2.5) under different prompting strategies compared with oral medicine experts using prospective, histopathology-verified clinical vignettes. In a prospective, paired diagnostic accuracy study, Gemini pro 2.5 (a multimodal LLM) was evaluated under three prompting strategies: Direct (P-1), Chain-of-Thought (P-2), and Self-Reflection (P-3) on 300 oral lesion cases with histopathologic confirmation. Each prompt was applied to identical inputs and compared against diagnoses from board-certified oral medicine specialists. Accuracy, rubric-based narrative quality, probability calibration, and computational efficiency were assessed under STARD-AI guidelines. Human experts achieved the highest Top-1 accuracy (61%), but Chain-of-Thought prompting (P-2) led AI performance in Top-3 accuracy (82%) and produced the highest explanation quality (mean rubric score 8.49/10). No AI prompt matched human performance in low-difficulty cases. P-2 also showed the best calibration (Brier score 0.238) compared to P-1 and P-3. Resource-wise, Direct prompting was fastest, but longer outputs modestly improved Top-3 recall. Mixed-effects modeling confirmed that AI performance varied significantly by prompt structure, highlighting context-specific trade-offs. Prompt structure significantly affects the diagnostic performance and interpretability of AI-generated differentials in oral lesion diagnosis. While expert clinicians remain superior in straightforward cases, structured prompting, particularly Chain-of-Thought, may enhance AI reliability in complex diagnostic scenarios. These findings support the integration of prompt engineering into AI-assisted diagnostic tools to augment clinical decision-making in oral medicine.

**Supplementary Information:**

The online version contains supplementary material available at 10.1038/s41598-025-22979-z.

## Introduction

Artificial intelligence (AI) is becoming a valuable tool in diagnostic medicine, especially with large language models (LLMs) that can process both text and images. Gemini Pro 2.5, developed by Google DeepMind, is one such model. It integrates clinical narratives and medical images and has shown expert-level performance in early evaluations^1^. Despite this potential, integrating AI into clinical workflows remains challenging due to concerns about transparency, generalizability, and interpretability. Many existing evaluations of AI systems rely on idealized or retrospective data that do not reflect the complexity of real-world clinical environments, limiting the transferability of such models into practice^1,2^.

The quality and reliability of AI model outputs in clinical settings are significantly influenced by prompt design. Structured prompts such as the SMART framework (Seeker, Mission, AI Role, Register, Targeted Question) have been shown to substantially enhance the accuracy, relevance, and completeness of responses generated by large language models (LLMs) in medical contexts. In a multicenter evaluation involving head and neck surgeons, SMART-structured prompts yielded significantly higher quality scores across all assessment dimensions compared to unstructured inputs, particularly improving source reliability and interpretability in clinical scenarios^3^. Similarly, iterative prompt engineering in real-time clinical deployments reduced negative sentiment and improved AI usability in electronic health record messaging^4^. These findings reinforce that carefully designed prompts not only optimize model accuracy but also enhance clinical communication and trustworthiness, emphasizing prompt engineering as a critical tool in AI-driven healthcare applications.

AI model performance in clinical tasks is highly sensitive to how prompts are designed, especially when operating on multimodal medical data. Prompt engineering, crafting tailored instructions to guide model output, has emerged as a critical method to enhance accuracy and interpretability in diagnostic applications. A study on GPT-4 Vision’s performance in medical imaging demonstrated that refined prompts could significantly improve diagnostic precision and relevance, especially for complex modalities like CT and MRI, by enabling more structured interpretive strategies^5^. Likewise, zero-shot prompt engineering achieved diagnostic accuracies exceeding 88% in a challenging liver tumor classification task using GPT-4 and BioGPT, suggesting that prompt design can rival traditional model fine-tuning in effectiveness^6^. However, few studies have prospectively and rigorously compared different prompting strategies on real patient cases using blinded expert evaluation and gold-standard histopathology. This study addresses that gap by examining how prompt structure impacts both diagnostic accuracy and the quality of reasoning in a real-world, expert-blinded clinical setting.

This is a prospective, paired diagnostic accuracy comparison between a multimodal LLM (Gemini Pro 2.5, Google DeepMind, Mountain View, CA) and experienced oral medicine specialists using the same set of lesion cases. We specifically investigated how different prompt engineering strategies, Direct querying, Chain-of-Thought reasoning, and Self-Reflection affect the model’s diagnostic performance and confidence calibration. The novelty of our approach lies in a within-subject design with blinded rubric scoring and inclusion of the full spectrum of diagnostic case difficulty. We assessed performance across multiple clinical metrics (accuracy, narrative quality, calibration, and latency), providing a rigorous benchmark for future AI diagnostic systems in visually dependent fields.

## Subjects and methods

### Study design

The study was conducted in compliance with the Declaration of Helsinki (2013 revision) and reported in accordance with STARD 2015 and its STARD-AI extension^7^. This is a prospective, paired diagnostic-accuracy study comparing a multimodal large-language model (Gemini Pro 2.5) with board-certified oral-medicine experts. Gemini Pro 2.5, selected for its ability to analyze clinical photographs alongside structured patient histories, was tested under three prompting strategies: (**P1**) direct prompt, (**P2**) chain-of-thought, and (**P3**) self-reflection. Each case served as its own control, enabling head-to-head comparison of the prompts’ diagnostic outputs against the expert reference diagnosis. The study complied with the Declaration of Helsinki (2013 revision). Ethical approval was granted by the Faculty of Dentistry, Ain Shams University Research Ethics Committee (FDA-SU-REC IR072502). Written informed consent was obtained for the use of de-identified clinical data and images.

### Setting

Cases were prospectively collected from the oral medicine clinics of three academic centers in Egypt: Ain Shams University, Cairo; King Salman International University, South Sinai; and Galala University, Suez, between January 1, 2024, and March 31, 2025. All patients underwent clinical examination and biopsy as part of standard care. For each case, a standardized intraoral photograph was taken using a high-resolution digital camera (Canon EOS 700D, Canon Inc., Tokyo, Japan) equipped with a Sigma 105 mm f/2.8 DG macro lens and Godox MF-R76 macro ring flash, under consistent background and lighting conditions. Images were stored in JPEG format at full resolution and were independently reviewed by two senior clinicians, with those showing blur, glare, or poor framing excluded. A brief vignette was also recorded for each case, summarizing clinical signs, symptoms, and relevant investigations. The study setting comprises tertiary care clinics, ensuring a mix of common and complex oral lesions.

### Sample size calculation

Sample-size estimation was informed by an internal, prospective 20-case pilot conducted by our team evaluating Gemini Pro 2.5’s Top-1 diagnostic accuracy across pre-stratified difficulty tiers (low/medium/high). Observed accuracy rates were approximately 68% for low-difficulty, 55% for medium-difficulty, and 49% for high-difficulty cases. Using the binomial proportion formula with a 95% confidence level (Z = 1.96) and a ± 10% margin of error, the required sample sizes were calculated as 84 cases for low-difficulty, 96 for medium-difficulty, and 96 for high-difficulty lesions. This yielded a total minimum of 276 cases. To allow for potential exclusions (e.g., poor image quality or ambiguous histology) and ensure robust subgroup analyses, we set a final recruitment target of 300 cases, approximately allocated as 85 low-, 105 medium-, and 110 high-difficulty lesions. Calculations were performed using OpenEpi version 3.01^8^, and verified in R version 4.3.1.

### Participants

We included consecutive adult patients (age ≥ 18 years) presenting with any oral lesion that had a definitive histopathological diagnosis (reference standard). Lesions were classified a priori into five diagnostic categories to reflect the breadth of oral pathology: malignant (e.g., oral squamous cell carcinoma, verrucous carcinoma, salivary-gland malignancies, lymphoma); benign (e.g., irritation fibroma, lipoma, pleomorphic adenoma, hemangioma); inflammatory/immune-mediated (e.g., oral lichen planus/lichenoid mucositis, mucous membrane pemphigoid); reactive/proliferative (e.g., traumatic ulcer, pyogenic granuloma, peripheral giant cell granuloma); and infectious (e.g., candidiasis, herpes simplex ulceration, HPV-related warts). Terminology followed standard oral pathology references and WHO/ICD-O where applicable. Inclusion required that a clinical photograph of the lesion be taken within 14 days before biopsy and that a concise clinical history be available. Cases were excluded if the images were of poor quality (e.g., significant blur, lighting glare, or framing errors) or if essential clinical information was missing.

### Clinical vignettes development

For each patient included, a senior clinician created a case vignette summarizing clinical history, symptoms, and oral findings and included relevant clinical and radiographic images. Vignettes followed a standardized ≤ 150-word template with fixed fields (age/sex; chief complaint & duration; anatomic site/laterality; size in mm, color, borders, surface; symptoms; temporal evolution; local irritants/trauma; risk factors tobacco/alcohol/HPV/immunosuppression; comorbidities/medications; regional nodes; relevant investigations), used neutral, non-leading wording, and recorded missing data explicitly as ‘unknown/not assessed.’ Each vignette was paired with standardized, de-identified intraoral photographs. Each scenario was saved as an individual file in Microsoft Word 2016 (Microsoft Corporation, Version 16.0, 64-bit,.docx, Office Open XML) on Windows 11, including the relevant clinical and, where applicable, radiographic images; files were then exported to PDF (PDF 1.7) via Word’s Save As, and PDFs were used for model ingestion. This standardized format ensured consistency for AI evaluation and expert comparison.

### Index tests (AI model and expert diagnosis)

#### AI model and prompting strategies

AI model and prompting strategies

The index test was the diagnosis generated by the multimodal LLM (Gemini Pro 2.5, Google DeepMind, Mountain View, CA) from each case’s clinical data (photo and vignette). Gemini Pro 2.5 is a state-of-the-art model capable of analyzing both textual and image inputs simultaneously. We accessed the model via the Gemini API (March 2025 version), using a deterministic setting (temperature = 0) to ensure output consistency. Three distinct prompt structures were designed to query the model for a differential diagnosis of each lesion.

- *Direct Prompt* (***P-1***): The model was instructed to output its top three diagnoses for the lesion along with an estimated probability for each (summing to 100%), without any intermediate reasoning. This serves as a baseline direct query.
- *Chain-of-Thought Prompt* (***P-2***): The model was cued with an instruction to “reason step by step” internally before giving the final diagnoses. It was asked to silently consider lesion features and then output only the top three diagnoses with probabilities, without showing its reasoning. Chain-of-thought prompting has been shown to significantly improve LLM performance on complex reasoning tasks^9^, and we hypothesized it would yield more accurate differentials by encouraging systematic analysis.
- *Self-Reflection Prompt* (***P-3***): The model was prompted to emulate an expert who double-checks their answer. It first had to give a single provisional diagnosis with a probability, then provide a brief self-critique of that choice, and finally either confirm the initial diagnosis or revise and list a three-item differential with probabilities. This technique of self-critique and revision is intended to let the model catch and correct its own errors, analogous to human reflective practice; such “self-reflection” approaches have been found to improve problem-solving accuracy in LLMs by identifying mistakes before finalizing answers^10^.

Each case’s photograph and vignette were provided to Gemini Pro 2.5 under each of the three prompt strategies in randomized order. We used a Latin-square design to rotate the prompt order across cases, mitigating any carryover or learning effects between prompts. The model was re-initialized (new session) for each prompt to prevent memory of prior outputs. All model queries were executed with identical settings aside from the prompt text. The LLM outputs specifically the differential diagnosis lists with probabilities that were recorded for analysis. The model’s Top-1 predicted diagnosis (highest probability choice) under each prompt was considered its primary answer for that prompt. “Top-3” performance was also assessed, meaning whether the correct diagnosis appeared anywhere in the model’s three suggestions.

#### Human expert comparator

Two board-certified oral medicine specialists (each with > 10 years’ experience) independently reviewed every lesion photograph and vignette. They were blinded to both the AI outputs and the histopathological diagnoses. Each expert listed up to three candidate diagnoses per case in descending order of likelihood, assigning probabilities that summed to 100%. From these, we extracted each expert’s Top-1 diagnosis and the set of diagnoses (with probabilities) for their Top-3 differential. Inter-rater agreement on the Top-1 diagnosis was excellent (Cohen’s κ = 0.82, 95% CI 0.74–0.90). For analyses requiring a single human reference standard, the two experts’ probability estimates were averaged for each diagnostic option, yielding one consensus probability distribution per case. Any differences in diagnostic labels were first reconciled through discussion to ensure the same terminology. This probability-averaged consensus served as the human benchmark, enabling a direct, like-for-like comparison with the model’s confidence-ranked outputs.

### Reference standard

The reference standard for final diagnosis was the histopathological diagnosis of the lesion, obtained from biopsy and examination of tissue by two certified oral pathologists. Histopathologic evaluation (tissue biopsy with microscopic analysis) is the gold standard for definitive diagnosis of oral lesions^11^, offering high accuracy even when clinical impressions are uncertain. For each case, the pathology report’s diagnosis was recorded and standardized using World Health Organization (WHO) International Classification of Diseases for Oncology (ICD-O) terminology to ensure consistency in naming. This reference standard was used to evaluate the correctness of the AI and expert diagnoses. The individuals interpreting the index tests (the AI model and the human expert) did not have access to the reference standard at the time of diagnosis, and those assessing study outcomes were blinded to the reference, as detailed below, to avoid incorporation bias.

### Case difficulty categorization

Before unblinding the results, we stratified the cases into three diagnostic difficulty tiers: Low, Medium, and High based on two senior clinicians’ independent judgment of each lesion’s complexity and subtlety. They considered factors such as lesion prevalence, clarity of clinical presentation, and known diagnostic pitfalls. Difficulty was defined based on clinical complexity across all lesion categories: (i) low: typical, well-demarcated, common lesions with pathognomonic features and minimal overlap; (ii) middle: moderately atypical lesions with overlapping features or diagnostic ambiguity requiring history integration; and (iii) high: rare, atypical, or ambiguous lesions with significant overlap or confounding factors. Discrepancies were resolved by consensus. These criteria were adapted from standard oral medicine and pathology references^12,13^ and a previously published framework on diagnostic ambiguity^14^. Initial difficulty assignments showed substantial inter-rater agreement (Cohen’s κ = 0.78, 95% CI 0.70–0.86). Any discordant ratings were resolved by discussion to reach a consensus. This categorization, conducted independently of the reference standard and blinded to all diagnostic outputs, was finalized before examining either AI or human performance. This ensured an unbiased, predefined subgroup analysis of accuracy across difficulty levels while mitigating spectrum bias.

**Ethics approval.** The study was conducted in compliance with the Declaration of Helsinki (2013 revision**)** and approved by the Ethical Committee of the Faculty of Dentistry, Ain Shams University (FDA SU-REC: IR072502)

**Consent to participate.** Informed consent was obtained for the use of de-identified case data.

## Outcome measures

The primary outcome was Top-1 diagnostic accuracy for each method: whether the first-ranked diagnosis from each index test (each AI prompt and the human expert benchmark) exactly matched the histopathology diagnosis. We also evaluated Top-3 accuracy, defined as whether the correct diagnosis was listed anywhere in the three-item differential.

Secondary outcomes captured diagnostic quality and efficiency:

- *Differential-quality rubric* (0–10). Two blinded oral pathologists Differential Diagnosis Quality (0–10 rubric score): Two blinded oral pathologists independently scored each AI-generated differential diagnosis on five criteria (correctness of the diagnoses, clinical plausibility, breadth of differential, logical use of probabilities, and clarity of terminology). Scores were assigned on a 0–2 scale for each criterion and summed (maximum 10). Large scoring discrepancies (≥ 3 points) were adjudicated through discussion, and the mean of the two scores was used for analysis.
- *Top-1 probability:* The explicit probability (confidence) that the AI model or human expert assigned to their Top-1 diagnosis, used for calibration analysis (e.g., calculation of Brier score).
- *Confidence-weighted recall @3 (CW-Recall):* the sum of the model’s assigned probabilities (out of 100%) for the correct diagnosis across its top three suggestions. This metric extends standard recall by incorporating confidence, rewarding not only inclusion of the correct label but also higher confidence in that label. CW-Recall has been used in prior AI diagnostic studies to capture both accuracy and calibration in a single measure^15^.
- *Brier score*: the mean squared error between predicted probabilities and the binary true outcome (correct vs. incorrect) across all cases, measuring calibration (lower is better)^16^.
- *Expected Calibration Error (ECE):* the weighted average difference between confidence and accuracy in discretized probability bins, with lower ECE indicating better alignment between predicted confidence and observed accuracy^17^.
- *Latency (ms) and output length (tokens)*: The computational time taken and the number of tokens (words or word pieces) generated for each output, recorded automatically for each query as proxies for efficiency and verbosity.
- *Inter-rater reliability* for rubric scoring, reported as Cohen’s κ.

Collectively, accuracy metrics gauge effectiveness; Top-1 probability and CW-Recall assess calibration; rubric scores capture explanatory quality; and latency/tokens reflect efficiency. AI outcomes were compared with the human benchmark wherever applicable.

### Data collection and test procedure

Intra-oral photos were de-identified, paired with a case vignette (age, sex, site, duration, risk factors), and fed to Gemini pro 2.5 under three prompt styles (Direct, Chain-of-Thought, Self-Reflection) in a Latin-square order (*representative cases and outputs of different prompt an expert responses are shown in* Supplementary Figure S1). Each run recorded the model’s full differential diagnosis output, the probabilities assigned, the output token count, and the API query latency. The model session was reset between prompts to prevent any cross-prompt information leakage.

Two board-certified oral-medicine specialists (human comparators), blinded to AI outputs and histopathology, reviewed the same set of photo-vignette cases and provided their differential diagnoses with probabilities totaling 100%. They achieved substantial agreement (κ = 0.82) on Top-1 diagnoses, and their probability estimates were averaged to form the consensus human diagnosis per case, as described above.

Rubric scoring of AI differentials was performed by two oral pathologist judges who were blinded to which prompt produced each output and to the true diagnosis. The pathologists providing the reference standard diagnoses were not involved in scoring or analysis. We implemented consecutive case inclusion to limit selection bias, Latin-square prompt randomization to prevent prompt-order effects, and anonymized coding of prompts during analysis to maintain blinding throughout the study workflow.

### Statistical analysis

Analyses were run in Python 3.9 (Statsmodels 0.13.5, SciPy 1.9.3), treating each case as a paired observation across four diagnostic methods (Expert, P-1, P-2, P-3). Inter-rater reliability for rubric scoring was excellent (Fleiss κ), and all tests used two-tailed α = 0.05 with Holm adjustment for multiplicity, reporting exact p-values, 95% CIs, effect sizes, and Bayes factors. Primary accuracy differences were screened with Cochran’s Q, followed when significant by pairwise McNemar tests; a two-one-sided-test (TOST) framework and Jeffreys matched-pairs Bayes factors evaluated non-inferiority of AI to the expert (Δ ± 5 pp). Accuracy dependence on case difficulty was probed via a mixed-effects logistic model (Prompt × Difficulty, random intercept by case) and stratified McNemar tests. Rubric scores were compared with Friedman and post-hoc Wilcoxon tests, complemented by a linear mixed-effects model. Calibration was quantified with Brier score, Expected Calibration Error, Hosmer–Lemeshow χ², and reliability curves, while efficiency metrics (latency, token count) were analyzed by repeated-measures ANOVA and correlated with accuracy. Finally, Bland–Altman plots and mixed-effects models contrasted the best AI prompt with the human benchmark, ensuring a comprehensive assessment of accuracy, explanation quality, calibration, and computational efficiency.

## Results

### Cohort characteristics

**Fig. 1:**
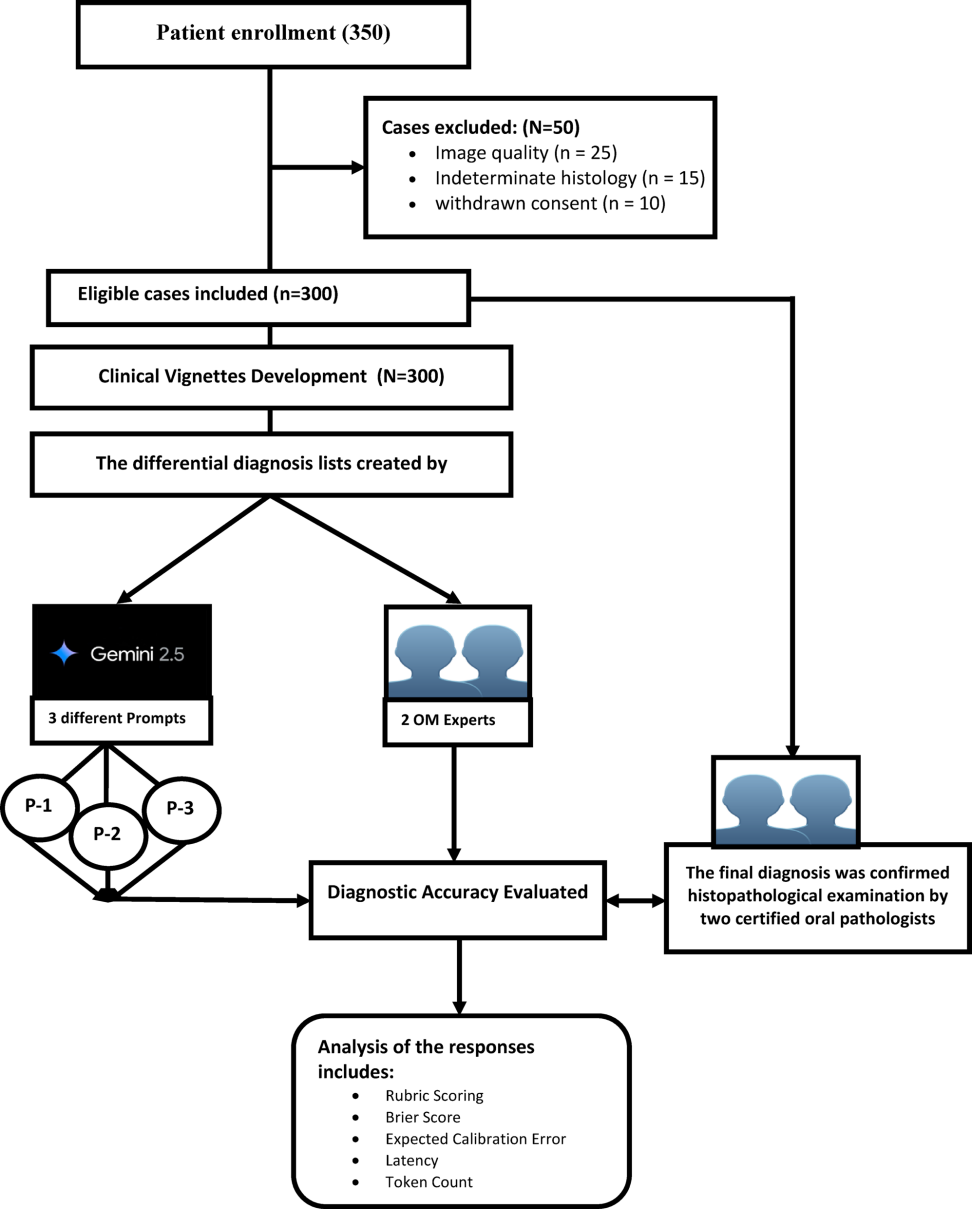
Study design.

Between 1 January and 31 March 2025, 350 consecutive cases met all inclusion criteria; 50 were excluded for image quality (*n* = 25), indeterminate histology (*n* = 15), or withdrawn consent (*n* = 10), leaving **300 lesions** for paired analysis (Fig. 1). These 300 cases were equally distributed across five diagnostic categories: malignant (*n* = 60), benign (*n* = 60), inflammatory (*n* = 60), reactive (*n* = 60), and infectious (*n* = 60). The final set comprised 84 low, 108 medium, and 108 high-difficulty lesions as pre-stratified in the Methods.

### Diagnostic accuracy

#### Overall performance

The Top-1 diagnostic accuracy was highest for the human experts at 61%, followed by the Chain-of-Thought prompt (P-2) at 55%, with the Direct (P-1) and Self-Reflection (P-3) prompts each at 49%. When considering Top-3 accuracy (allowing credit if the correct diagnosis appeared anywhere in the differential), all methods improved: P-2 achieved the highest Top-3 accuracy at 82%, followed by the human experts at 76%, P-3 at 73%, and P-1 at 70%. A Cochran’s Q test found no significant global difference across the four diagnostic methods for either Top-1 accuracy (χ² = 2.87, *p* = 0.41) or Top-3 accuracy (χ² = 3.00, *p* = 0.39). Pairwise McNemar tests between each method pair were likewise non-significant after Holm correction (Table 1 for detailed values).

**Table 1:** Cochran’s Q and McNemar Test Results for Diagnostic Accuracy of three prompt (Top-1, and Top-3)

Level		Comparison	Statistic	χ² Statistic	*P*-value	Bonferroni *p*-value
Top-1		P1 vs. P2 vs. P3 vs. Expert	CochranQ	2.87	0.4122 ns	–
Top-3		P1 vs. P2 vs. P3 vs. Expert	CochranQ	3	0.3916 ns	–
Top-1		Expert vs. P1	McNemar	3	0.3438 ns	1
		Expert vs. P2	McNemar	4	0.7539 ns	1
		Expert vs. P3	McNemar	3	0.3438 ns	1
		P1 vs. P2	McNemar	2	0.6875 ns	1
		P1 vs. P3	McNemar	2	1.0000 ns	1
		P2 vs. P3	McNemar	2	0.6875 ns	1
Top-3		Expert vs. P1	McNemar	3	0.7266 ns	1
		Expert vs. P2	McNemar	1	0.6250 ns	1
		Expert vs. P3	McNemar	2	1.0000 ns	1
		P1 vs. P2	McNemar	0	0.1250 ns	0.75
		P1 vs. P3	McNemar	4	1.0000 ns	1
		P2 vs. P3	McNemar	1	0.3750 ns	1

*Significant: *p* < 0.05 (before Bonferroni adjustment) ns = not significant

#### Difficulty subgroups

Accuracy declined with increasing case difficulty for all methods (Table 2). In high- and medium-difficulty cases, differences between the AI prompts and humans were not statistically significant. However, in low-difficulty (straightforward) cases, human experts outperformed all AI prompts at the Top-1 level (89% vs. 43–68% among the prompts; *p* = 0.044 for the difference between the human benchmark and the best AI prompt). No significant prompt-specific differences were observed in Top-3 accuracy within any difficulty tier.

**Table 2:** Diagnostic Performance by Difficulty Level: ChatGPT, Gemini, and Human Expert Accuracy Across Top-1, and Top-3

Diagnostic Difficulty	Top-1				*P*-value	Top-3				*P*-value
	Experts	*P*−1	*P*−2	*P*−3		Experts	*P*−1	*P*−2	*P*−3	
High (108)	27 (25%)	36 (33%)	36 (33%)	27 (25%)	0.88 ns	45 (42%)	54 (50%)	54 (50%)	45 (42%)	0.84 ns
Middle (108)	81 (75%)	72 (67%)	72 (67%)	72 (67%)	0.90 ns	99 (92%)	81 (75%)	108 (100%)	99 (92%)	0.28 ns
Low (84)	75 (89%)	36 (43%)	57 (68%)	48 (57%)	0.044*	84 (100%)	75 (89%)	84 (100%)	75 (89%)	0.57 ns

*p < 0.05. **ns** = Not significant.

#### Non-inferiority analyses

Non-inferiority analyses (Δ = ±5% points) showed that none of the prompting strategies were statistically inferior to one another at the predefined margin. Chain-of-Thought prompting (P-2) achieved a modest but favorable mean difference in Top-1 accuracy compared to Direct prompting (P-1) (+ 0.06; 95% CI − 0.11 to + 0.24; discordant counts 2/4). Self-Reflection (P-3) performed similarly to Direct prompting (− 0.03; 95% CI − 0.22 to + 0.15; discordant counts 2/2). When P-3 was compared against P-2, the mean difference was − 0.09 (95% CI − 0.28 to + 0.10; discordant counts 4/2), again within the non-inferiority margin. Bayes factors provided moderate to strong evidence in favor of non-inferiority for P-2 vs. P-1 (BF₁₀ = 9.14), weaker support for P-3 vs. P-1 (BF₁₀ = 3.20), and strong evidence for P-3 vs. P-2 (BF₁₀ = 9.14). Equivalence (two one-sided tests) was not achieved for any pair, as the confidence intervals crossed the ± 5% boundaries.

### Rubric quality and narrative

Rubric-based evaluation of the diagnostic explanations revealed a significant effect of prompt strategy on narrative quality (Friedman χ² = 14.35, *p* < 0.001; (Table 3). The Chain-of-Thought prompt (P-2) produced the highest mean rubric score (8.49 ± 1.58 out of 10), outperforming the Direct prompt (P-1) by an average of 0.88 points (Wilcoxon signed-rank test, adjusted *p* = 0.0005). P-2 also scored higher than Self-Reflection (P-3) by 0.34 points on average, although this difference was not statistically significant (adjusted *p* = 0.31). Analysis of rubric scores showed a significant main effect of prompting strategy (χ² = 16.11, df = 6, *p* = 0.013), indicating that the type of prompt influenced the quality of generated differentials. By contrast, case difficulty alone did not have a significant effect (χ² = 9.68, df = 6, *p* = 0.139), nor was there a significant Prompt × Difficulty interaction (χ² = 4.32, df = 4, *p* = 0.365). These results suggest that the prompting strategy, rather than lesion difficulty, was the primary driver of variation in rubric-based explanation quality.

**Table 3:** Rubric-Score differences across prompts (Friedman omnibus + Wilcoxon).

Test	Comparison	W (statistic)	Raw *p*	Holm-adjusted *p*
**Friedman χ²**	P-1 vs. P-2 vs. P-3	**14.35**	***p*** < 0.001*	—
Wilcoxon	**P-1 vs. P-2**	57.0	0.00018	0.00054*
	P-1 vs. P-3	122.0	0.0587	0.1174
	P-2 vs. P-3	55.5	0.3104	0.3104

*Significant at *p* < 0.05. ns = not significant.

### Calibration and reliability

**Fig. 2:**
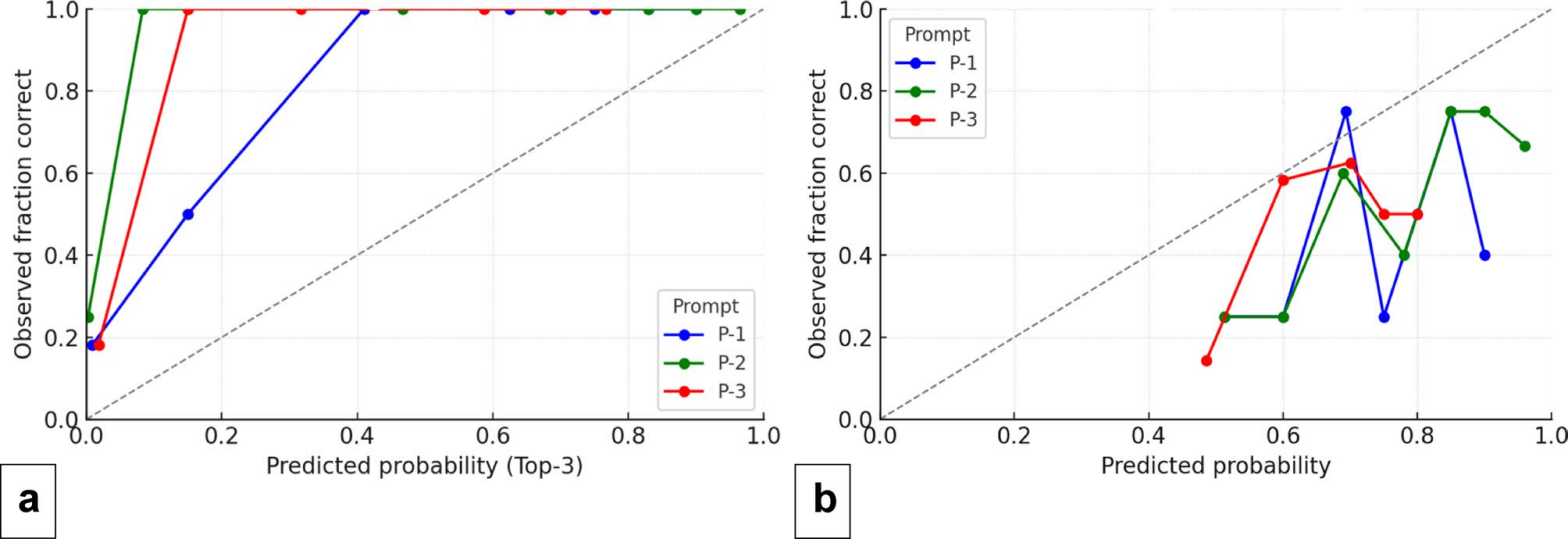
Reliability curves for Top-3 (**a**) and Top-1 (**b**) predictions, showing the observed fraction correct versus predicted probability for P-1 (blue), P-2 (green), and P-3 (red).

The calibration of predicted probabilities varied by prompt. Chain-of-Thought prompting (P-2) showed the most favorable calibration among the AI methods, with the lowest Brier score (0.238) and lowest ECE (0.247), indicating its confidence estimates were closest to ideal. The Direct prompt (P-1) had the poorest calibration (Brier = 0.291, ECE = 0.279), while Self-Reflection (P-3) was intermediate (Brier = 0.258, ECE = 0.258). For comparison, the human consensus benchmark was better calibrated overall than the AI, with a Brier score of 0.152 and ECE of 0.252. Reliability curves (Fig. 2) reflected these findings: P-1’s predictions were over-confident (curve bowed above the diagonal), P-3 was slightly under-confident (curve below the diagonal), and P-2’s curve tracked closest to the perfect calibration line. A generalized estimating equation analysis found no significant Prompt × Difficulty interaction on calibration slopes (χ² = 0.49, *p* = 0.78), suggesting each prompt’s calibration performance was consistent across different case difficulties. (Formal statistical comparison of Brier or ECE between prompts was not performed, so these observations remain descriptive.)

In terms of confidence-weighted recall, at the strict Top-1 level (equivalent to weighting the correctness of a diagnosis by its assigned probability), we found all pairwise prompt comparisons to be statistically significant after Holm correction: P-2 > P-1, P-2 > P-3, and P-1 > P-3 (Table 4). However, at the Top-3 level of confidence-weighted recall, no significant pairwise differences emerged, as each method tended to assign some probability mass to the correct diagnosis when it was included in the top three.

**Table 4:** Confidence-Weighted recall at top-1 and top-3.

CW-Recall Level	Test	Comparison	Statistic†	Raw *p*	Holm-adj *p*
@1	Friedman χ²(2)	P-1, P-2, P-3	22.21	< 0.0001*	—
	Wilcoxon	P-1 vs. P-2	W = 34.5	0.0148 ns	0.0303*
		P-1 vs. P-3	W = 31.0	0.0303 ns	0.0303*
		P-2 vs. P-3	W = 32.5	0.0117 ns	0.0351*
@3	Friedman χ²(2)	P-1, P-2, P-3	14.28	< 0.0001*	—
	Wilcoxon	P-1 vs. P-2	W = 101.5	0.0601 ns	0.0601 ns
		P-1 vs. P-3	W = 109.0	0.0540 ns	0.1081 ns
		P-2 vs. P-3	W = 76.0	0.0198 ns	0.1081 ns

“†””Friedman rows report χ²; Wilcoxon rows report signed-rank statistic *W*.

* = significant after Holm correction; ns = not significant. Sig. (α = 0.05).

### Computational efficiency

The prompting strategies differed markedly in computational resource use (Table 1). Direct prompting (P-1) was the fastest, with an average latency of 34.7 ± 8.4 ms, and produced the shortest outputs (approximately 72 ± 9 tokens on average). Chain-of-Thought (P-2) generated roughly twice as much text (152 ± 36 tokens) and had the longest latency (55.5 ± 9.7 ms), while Self-Reflection (P-3) was intermediate in both output length and speed. A repeated-measures ANOVA confirmed significant variation in latency among the three prompt types (F = 45.28, *p* < 0.00001).

Despite these efficiency differences, longer runtime or output did not correspond to higher accuracy. In a mixed-effects logistic model that included latency and token count as covariates (for each case-prompt pair), neither latency nor tokens significantly predicted Top-1 accuracy. For Top-3 accuracy, latency again showed no effect on performance (OR = 1.007 per ms, 95% CI 0.96–1.05, *p* = 0.78). A one-standard-deviation increase in output length (approximately + 38 tokens) was associated with a modest increase in the odds of including the correct diagnosis in the top three (OR = 1.032, 95% CI 1.00–1.06, *p* = 0.046) (Table 5). In practical terms, more verbose explanations conferred a slight benefit for capturing the correct diagnosis somewhere in the differential, whereas producing answers faster did not trade off accuracy.

**Table 5:** Mixed-Effects Model — Odds ratios for tokens & latency (Top-1 and Top-3).

Term	Top-1				Top-3			
	OR	95% CI	*p*	OR per 1 SD▲	OR	95% CI	*p*	OR per 1 SD▲
Prompt P-2 vs. P-1	0.46	0.05–4.18	0.493	—	0.22	0.01–4.15	0.311	—
Prompt P-3 vs. P-1	1.01	0.13–7.81	0.993	—	11.23	0.76–166.9	0.079	—
Difficulty Low vs. High	1.51	0.26–8.92	0.650	—	8.67	0.87–85.9	0.065	—
Difficulty Mid vs. High	3.88	0.71–21.2	0.118	—	3.65	0.58–22.9	0.168	—
Tokens-out (per token)	1.007	0.99–1.03	0.500	1.58	1.032	1.00–1.06	0.046*	7.82
Latency-s (per s)	1.000	0.96–1.04	0.977	1.03	1.007	0.96–1.05	0.775	1.30

▲ *OR per 1 SD* reflects the odds-ratio for a one-standard-deviation increase in the numeric predictor (Top-1/Top-3):.

• tokens_out : SD ≈ 38 tokens ⇒ OR ≈ 1.58 (Top-1)/7.82 (Top-3).

• latency_ms : SD ≈ 9 s ⇒ OR ≈ 1.03 (Top-1)/1.30 (Top-3).

#### Summary of Trade-offs

Each prompt strategy showed advantages and drawbacks (Table 1). P-1 (Direct) was computationally lean and fastest, but it had the worst calibration and the lowest explanation quality. P-2 (Chain-of-Thought) provided the richest and best-calibrated narratives, at the cost of increased response time and verbosity. P-3 (Self-Reflection) offered intermediate performance on most metrics. No single prompting method was superior across all domains, underscoring that the optimal prompt may depend on the clinical context and priorities (e.g., speed vs. interpretability).

### Human vs. LLM comparison

**Fig. 3:**
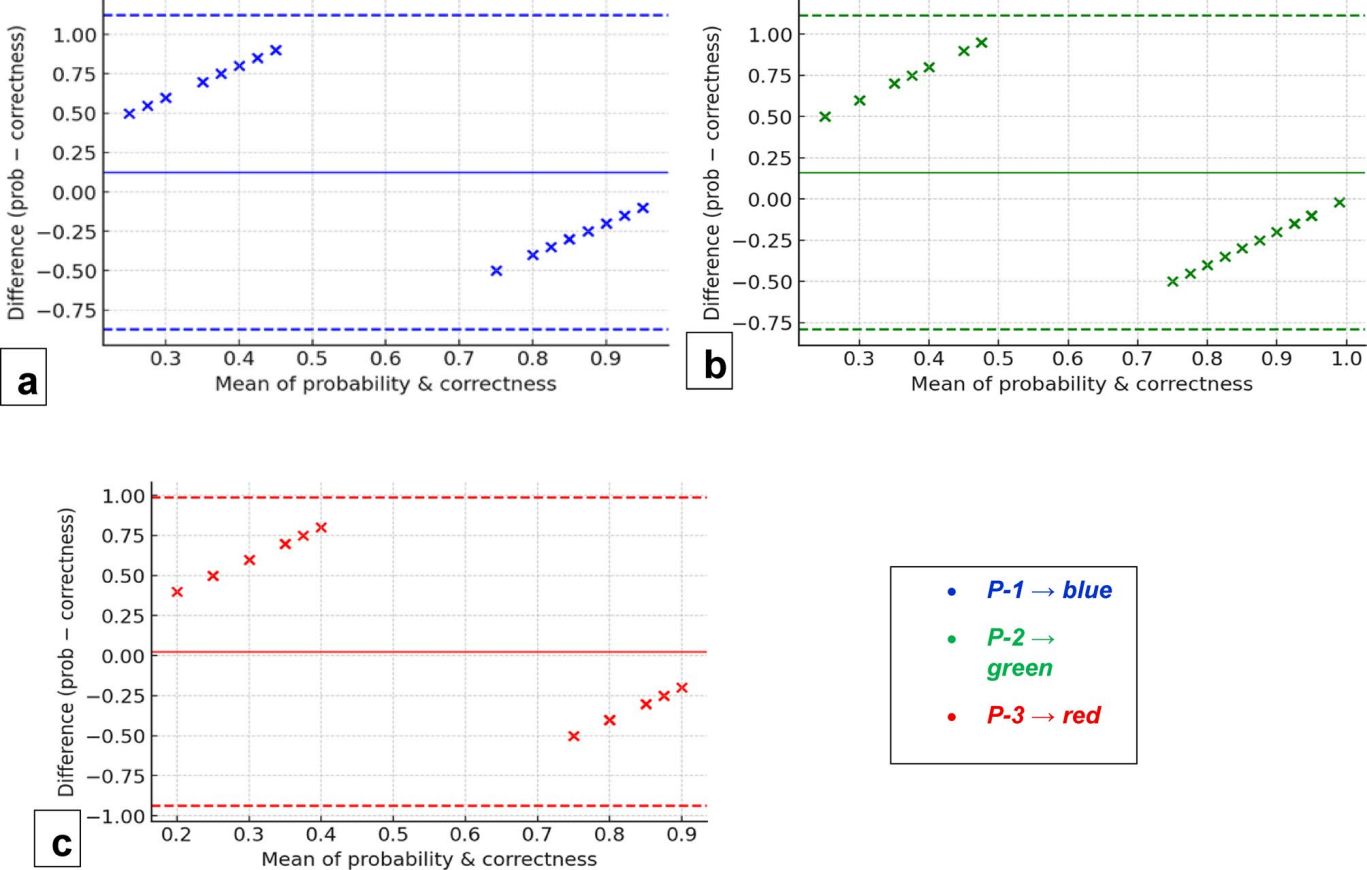
Bland-Altman plots comparing predicted probabilities; (***a***) (P-1) to Human correctness, (***b***) (P-2) to Human correctness, and (***c***) (P-3) to Human correctness.

In a direct comparison between the AI and human reference standard, a mixed-effects model pooling all prompts found no significant overall accuracy gap (odds ratio for AI vs. human = 0.74, 95% CI 0.44–1.23, *p* = 0.24). However, prompt-specific contrasts revealed a more nuanced picture: P-2 and P-3 each had significantly lower odds of a correct diagnosis than the human benchmark (OR 0.60, *p* = 0.006 for P-2; OR 0.67, *p* = 0.022 for P-3), whereas P-1’s accuracy did not differ statistically from the human (*p* = 0.30). This suggests that while structured prompts (P-2, P-3) improved the AI’s interpretability and Top-3 coverage, they did not surpass human diagnostic precision in Top-1 outcomes. Bland–Altman analysis (Fig. 3) showed that all AI prompts tended to overestimate their confidence in the correct diagnosis compared to the human benchmark’s actual accuracy, but these biases were relatively small and the limits of agreement encompassed zero.

## Discussion

To our knowledge, this is among the first prospective, expert-blinded benchmarks of different prompting strategies in a real-world diagnostic context. Our key finding is that the prompting strategy is not a trivial design choice but a core determinant of both diagnostic accuracy and narrative quality. Although human experts achieved the highest Top-1 performance (61%), the Chain-of-Thought prompt (P-2) was a close second (55%) and achieved the best Top-3 accuracy (82%, slightly above the human benchmark’s 76%). No overall accuracy differences emerged across methods, but human superiority was most evident in low-difficulty cases.

These findings complement prior evidence that structured prompts improve model reasoning^9,10,18^, and align with studies demonstrating that AI can match or even outperform humans in specific imaging tasks (e.g., liver tumors and chest X-rays) under the right conditions^19,20^. However, our results temper overly optimistic claims by emphasizing that an AI model’s relative performance varies by prompt type and case complexity. Similarly, studies in head and neck surgery using SMART prompt structures found that tailored prompts significantly improved accuracy, relevance, and clarity of AI responses^3^.

One possible mechanism is that Chain-of-Thought prompts simulate stepwise clinical reasoning, facilitating better performance on complex cases by guiding the model through analytic reasoning pathways akin to dual-process (System 2) cognition. In contrast, the lower Top-1 performance in easy cases may reflect the model’s lack of clinical intuition or an overelaboration that causes it to miss obvious diagnoses where human experts excel through rapid pattern recognition.

Evaluation of the explanations further underscored the benefits of structured prompting. Chain-of-Thought prompting (P-2) yielded clearly superior explanation quality, significantly outperforming Direct prompts (P-1) and marginally surpassing Self-Reflection (P-3) in our rubric scores. This advantage persisted across difficulty levels, indicating that structured reasoning enhances not only diagnostic accuracy but also the interpretability and plausibility of AI-generated outputs under varied conditions. These findings reinforce prior evidence that prompt design can shape the depth and clarity of AI responses in clinical settings^5,9^. Comparable gains in explainability have also been reported when prompts elicit stepwise or reflective reasoning strategies in multimodal diagnostic tasks^6^. A likely mechanism is that Chain-of-Thought prompts reduce ambiguity by encouraging the model to articulate intermediate reasoning steps internally, thereby producing explanations that mirror clinical logic. This emulates how human experts explain their decisions when teaching or documenting reasoning, enhancing clarity and justifiability, particularly in complex or uncertain cases.

Prompting strategies also differed substantially in computational resource demands, with Direct prompting (P-1) being the fastest and most concise, while Chain-of-Thought (P-2) had the longest latency and highest token count. Interestingly, despite these variations, output speed did not predict diagnostic accuracy for either Top-1 or Top-3 performance. However, longer narrative length modestly increased the likelihood of capturing the correct diagnosis in the Top-3 differential. This supports prior observations that verbosity, when used constructively, can enhance diagnostic completeness^5^. It also aligns with usability studies suggesting that clinicians value AI explanations more when they are sufficiently detailed, even at the expense of brevity^3,21^. One explanation is that longer outputs provide more opportunity for diagnostic breadth, allowing the model to surface rarer or alternative hypotheses that might be omitted in a very short response. This reflects how expert clinicians often use more extensive narratives to hedge uncertainty or consider atypical presentations, enhancing safety in diagnostic reasoning^5,9^.

Moreover, the incorporation of explanation-centered frameworks, as in the use of SHAP and LIME for model transparency^22^, highlights the increasing importance of interpretable AI in clinical workflows. Mechanistically, this suggests that prompts function as cognitive scaffolds guiding the LLM to articulate reasoning paths that align more closely with how clinicians approach differential diagnosis, thereby enhancing trust and usability.

When comparing AI performance to human experts, our mixed-effects analysis revealed no significant overall accuracy gap, but prompt-specific analyses painted a more nuanced picture. Both Chain-of-Thought (P-2) and Self-Reflection (P-3) had significantly lower odds of correctness compared to the human benchmark, while Direct prompting (P-1) did not differ statistically. This suggests that while structured prompts improve interpretability and Top-3 coverage, they do not necessarily boost an LLM’s Top-1 diagnostic precision beyond what can be achieved with a straightforward prompt. These findings are consistent with recent studies indicating that LLMs, though capable of impressive reasoning, still trail clinical experts in real-world diagnostic performance, especially for straightforward cases or when ground truth is pathology-confirmed^23,24^. The slight overestimation of confidence we observed across prompts may reflect a known calibration issue in LLMs, where linguistic fluency and high token probabilities can be mistaken for diagnostic certainty^22^. This underlines the importance of maintaining expert oversight in deployment and incorporating calibration techniques into clinical AI systems.

Importantly, while this study tested a state-of-the-art model (Gemini Pro 2.5) under deterministic prompting conditions, it remains unclear how generalizable these findings are to other models, future LLM versions, or real-time use. Furthermore, structured prompting remains an experimental interface; its feasibility in busy clinical workflows, including time demands, training, and system integration, warrants dedicated evaluation. These translational challenges highlight the need for implementation science alongside model development to ensure responsible AI integration.

These findings have direct clinical implications. Incorporating structured prompts like Chain-of-Thought could improve explanation clarity and differential breadth, potentially aiding decision-making in complex or ambiguous cases^5,9^. From a policy perspective, our results advocate for prompt standardization in clinical AI tools, aligning with emerging frameworks on responsible AI implementation in healthcare^24^. Theoretically, our study also refines dual-process models of diagnostic reasoning by demonstrating that LLMs guided by analytic prompts (akin to System 2 thinking) produce more coherent and calibrated outputs supporting the idea that reasoning structure can be externally induced in non-conscious systems. This opens new avenues for research on how prompt engineering can operationalize cognitive models within medical AI.

This study’s strengths include a rigorously controlled, within-subject design using a (300) real-world, histopathology-validated dataset and expert human benchmarks, ensuring high external validity. The prospective, blinded comparison across three prompting strategies provides novel insight into how prompt design affects diagnostic performance, interpretability, and calibration. Additionally, the use of objective rubric-based scoring and appropriate mixed-effects modeling enhances the robustness and reproducibility of the findings.

### Limitations

Despite the study’s strengths, several limitations warrant consideration. First, although a cohort of 300 cases ensured adequate power for statistical testing, it may still constrain the applicability of our findings to other clinical settings or specialties. Second, all vignettes comprised static image-text pairs; therefore, the model’s performance in dynamic, real-time patient encounters remains untested. Third, evaluations were restricted to a single multimodal LLM Gemini Pro 2.5 queried under deterministic settings, limiting insight into how alternative architectures or temperature parameters might behave in practice. Finally, even with a standardized rubric and strong inter-rater agreement, aspects of narrative-quality assessment inevitably retain a degree of subjectivity.

### Future prospective

Future research should explore the generalizability of these findings across other specialties and diagnostic modalities, including dynamic, multi-turn clinical interactions. Comparative studies involving different LLM architectures and real-time clinician AI collaboration could further clarify how prompt design impacts usability and trust. Moreover, integrating formal explainability frameworks with prompt engineering may enhance both transparency and safety. Longitudinal studies assessing the impact of structured prompts on clinical decision-making, patient outcomes, and workflow integration will be essential for informing responsible AI deployment in healthcare.

## Conclusion

In summary, this study demonstrates that prompt design significantly influences the diagnostic accuracy, narrative quality, and clinical plausibility of AI outputs in multimodal oral lesion diagnosis. Structured strategies like Chain-of-Thought prompting improved both the interpretability of AI-generated explanations and the alignment between model confidence and clinical reasoning. While human experts still outperformed the AI model in straightforward diagnostic cases, the findings highlight prompt engineering as a critical, yet underutilized, tool in optimizing AI performance for clinical use. These insights underscore the growing clinical applicability of AI in diagnostic systems and emphasize the need for further integration of such models into real-world workflows, ensuring they remain interpretable, trustworthy, and aligned with the practical needs of healthcare professionals.

## Supplementary Information

Below is the link to the electronic supplementary material.

Supplementary Material 1

## Data Availability

Research data supporting this publication is available from the corresponding author upon request.
