# Supplementary figures and images for: Prompt-dependent performance of multimodal AI model in oral diagnosis: a comprehensive analysis of accuracy, narrative quality, calibration, and latency versus human experts
Source: Sci Rep. 2025 Oct 30;15:37932. doi: 10.1038/s41598-025-22979-z (PMC12575769; doi:10.1038/s41598-025-22979-z)

**Supplementary Figure S1. Case Examples with LLM Prompts and Expert Responses**


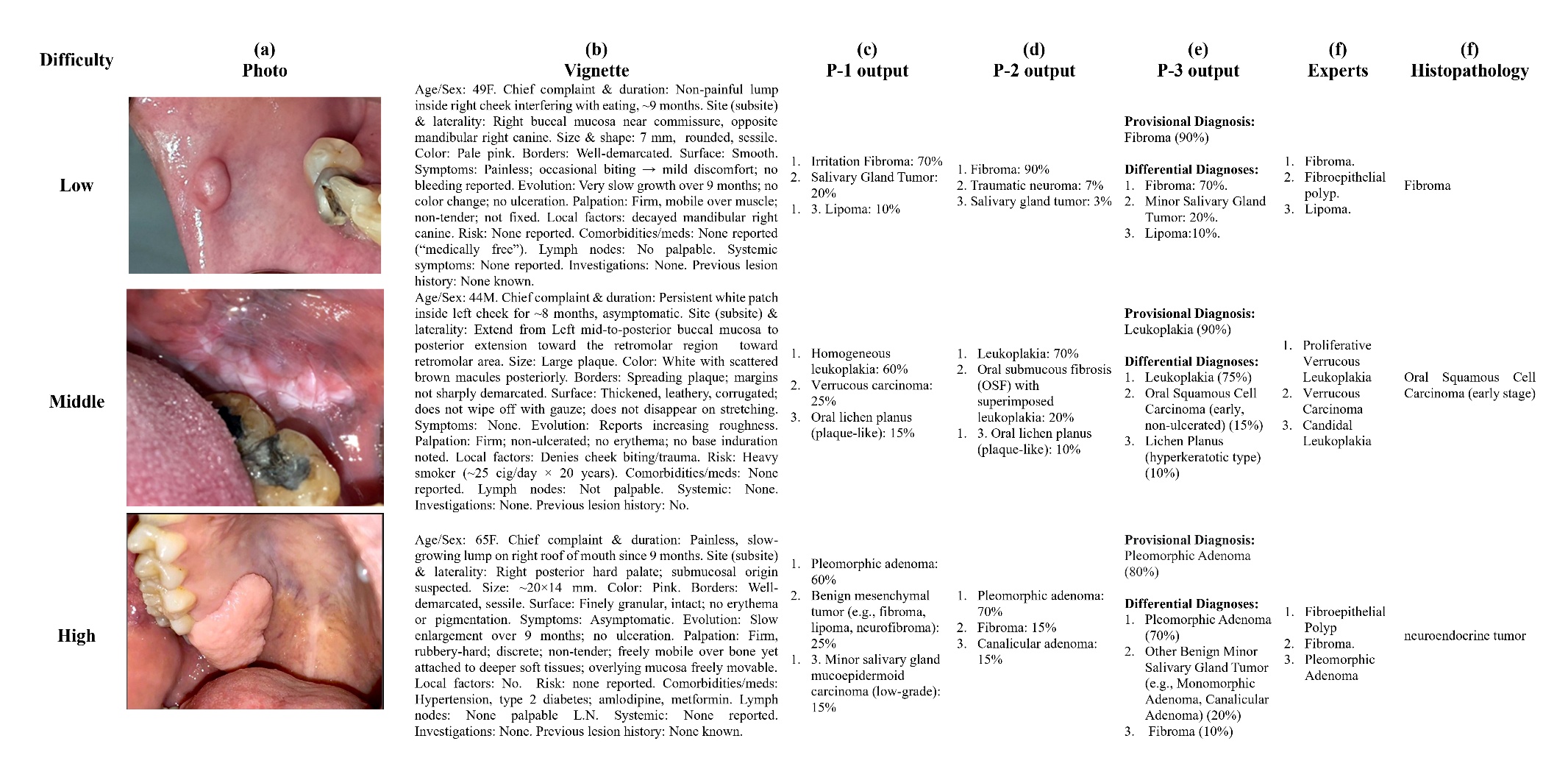

Supplement: Supplementary file 1 — Supplementary Material 1 [file 41598_2025_22979_MOESM1_ESM.docx]
